# The novel insecticides flupyradifurone and sulfoxaflor do not act synergistically with viral pathogens in reducing honey bee (*Apis mellifera*) survival but sulfoxaflor modulates host immunocompetence

**DOI:** 10.1111/1751-7915.13673

**Published:** 2020-09-28

**Authors:** Yahya Al Naggar, Robert J. Paxton

**Affiliations:** ^1^ General Zoology Institute for Biology Martin Luther University Halle‐Wittenberg Hoher Weg 8 Halle (Saale) 06120 Germany; ^2^ Zoology Department Faculty of Science Tanta University Tanta 31527 Egypt

## Abstract

The decline of insect pollinators threatens global food security. A major potential cause of decline is considered to be the interaction between environmental stressors, particularly between exposure to pesticides and pathogens. To explore pesticide–pathogen interactions in an important pollinator insect, the honey bee, we used two new nicotinic acetylcholine receptor agonist insecticides (nACHRs), flupyradifurone (FPF) and sulfoxaflor (SULF), at sublethal and field‐realistic doses in a fully crossed experimental design with three common viral honey bee pathogens, *Black queen cell virus* (BQCV) and *Deformed wing virus* (DWV) genotypes A and B. Through laboratory experiments in which treatments were administered singly or in combination to individual insects, we recorded harmful effects of FPF and pathogens on honey bee survival and immune gene expression. Though we found no evidence of synergistic interactions among stressors on either honey bee survival or viral load, the combined treatment SULF and DWV‐B led to a synergistic upregulation of *dicer‐like* gene expression. We conclude that common viral pathogens pose a major threat to honey bees, while co‐exposure to these novel nACHR insecticides does not significantly exacerbate viral impacts on host survival in the laboratory.

## Graphical abstract

Through laboratory experiments in which treatments were administered singly or in combination to individual insects, we recorded harmful effects of FPF and pathogens on honey bee survival and immune gene expression. Though we found no evidence of synergistic interactions among stressors on either honey bee survival or viral load, the combined treatment SULF and DWV‐B led to a synergistic up‐regulation of dicer‐like gene expression. We conclude that common viral pathogens pose a major threat to honey bees whilst co‐exposure to these novel nACHR insecticides does not significantly exacerbate viral impacts on host survival in the laboratory.

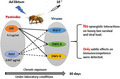

## Introduction

Honey bees (*Apis mellifera*) play an invaluable role in crop and wild plant pollination (Gallai *et al*., 2009; Moritz *et al*., 2010; Al Naggar *et al*., 2018). Europe and the United States have seen major overwinter losses of honey bee colonies since 2006, with multiple biotic and abiotic factors having been proposed in causing these colony declines (Jacques *et al*., 2017; Kulhanek *et al*., 2017; Gray *et al*., 2019; Neov *et al*., 2019). Though there is considerable support for the view that viral infection, particularly by *Deformed wind virus* (DWV) associated with varroa (*Varroa destructor*) ectoparasitic mite transmission, is a leading cause for elevated overwinter colony mortality (Genersch *et al*., 2010; Dainat *et al*., 2012; Francis *et al*., 2013; McMahon *et al*., 2016; Natsopoulou *et al*., 2017), scientific consensus is that exposure to multiple stressors (microbial infections, exposure to pesticides, loss of habitat and improper beekeeping practices) underlies honey bee colony losses (Dechaume Moncharmont *et al*., 2003; Potts *et al*., 2010; Gill *et al*., 2012; Vanbergen *et al*., 2013; Goulson *et al*., 2015; Manley *et al*., 2015; Jacques *et al*., 2017).

Pesticides are well‐known contributors to declining bee health (Dechaume Moncharmont *et al*., 2003; Desneux *et al*., 2007; Gill *et al*., 2012; Osborne, 2012; Al Naggar *et al*., 2015; Siviter *et al*., 2018b; Al Naggar and Baer, 2019). Here, we explore honey bee reactions to two new plant protection products: sulfoxaflor (SULF) and flupyradifurone (FPF), belonging to the chemical groups sulfoximines and butenolides, respectively. These insecticides, which share their mode of action with neonicotinoids as selective agonists of Nicotinic Acetyl Choline Receptors (NAChRs) (Zhu *et al*., 2011; Sparks *et al*., 2013), are a more recent entry to the insecticide market. Currently, FPF (Sivanto™ prime) and SULF (Transform^®^) are, respectively, approved for use in more than 30 and 81 countries worldwide, with effects on insect pollinators potentially comparable to those of neonicotinoid insecticides (Siviter *et al*., 2018a, 2020b).

Chronic exposure to SULF at field‐realistic concentrations has been shown to reduce egg laying and impair reproductive success in bumble bees (Siviter *et al*., 2018a, 2020b), though acute exposure to SULF did not affect their learning and memory (Siviter *et al*., 2019), or the escape response of locusts (Parkinson *et al*., 2020). Exposure of *A. mellifera* colonies to SULF in a flight enclosure caused acute toxicity but did not otherwise impact flight activity or long‐term colony development (Cheng *et al*., 2018). Exposure to high and non‐field‐realistic FPF dosages has been shown to affect the sensory (taste), cognition and motor abilities of honey bees (Hesselbach and Scheiner, 2018, 2019). At field‐realistic, worst‐case FPF doses, a significant reduction in olfactory associative learning performance has also been observed in the closely related bee species *Apis cerana* (Tan *et al*., 2017), and more subtle effects of FPF on *A. mellifera* have also been demonstrated in combination with factors such as bee age, seasonality, nutritional stress and exposure to other chemicals (Tong *et al*., 2019; Tosi and Nieh, 2019). Hesselbach *et al*. (2020) recently showed that chronic exposure to FPF can lead to the premature onset of foraging in *A. mellifera*. Additionally, exposure to field rates of Sivanto™ (FPF) and Transform^®^ (SULF) has also recently been shown to increase oxidative stress and induce apoptosis in *A. mellifera* (Chakrabarti *et al*., 2020). Studies have yet to investigate interactions between sublethal doses of these two novel pesticides and viral pathogens that affect honey bee health.

Another important cause of honey bee colony losses is the presence of the ectoparasitic mite *V. destructor*, which has spread worldwide, with significant impacts on honey bee colony health as a consequence of its transmission of a cocktail of viruses while feeding on honey bee haemolymph and fat bodies (Gisder *et al*., 2009; Mockel *et al*., 2011; Martin *et al*., 2012; Erban *et al*., 2015; Wilfert *et al*., 2016; Ramsey *et al*., 2019). A highly prevalent and relatively virulent virus transmitted by *V. destructor* and impacting honey bee colony health worldwide is *Deformed wing virus* (DWV), high titres of which cause developmental deformities and premature ageing in honey bees (Mockel *et al*., 2011; Natsopoulou *et al*., 2016; Tehel *et al*., 2019), and which lead to high overwintering colony losses (Highfield *et al*., 2009; Genersch *et al*., 2010; Dainat *et al*., 2012; Francis *et al*., 2013; McMahon *et al*., 2016; Natsopoulou *et al*., 2017). Recently, a genotypic variant of *Deformed wing virus* (DWV), genotype B (DWV‐B), has been shown to be more virulent than the original DWV genotype A (DWV‐A) in adult honey bees (McMahon *et al*., 2016, see also Gisder *et al*., 2018). In a follow‐up study using the same viral inocula, the same pattern of differential virulence was found between genotypes, but differences in virulence were not statistically significant (Tehel *et al*., 2020). Another widespread honey bee virus is *Black queen cell virus* (BQCV), which has been found in collapsing colonies (Mondet *et al*., 2014) and is potentially very virulent when gaining access to an adult honey bee’s haemocoel (Al Naggar and Paxton, 2020). BQCV also kills developing queen larvae, whose necrotic remains stain their pupal cells black (Spurny *et al*., 2017).

Scientific attention has been focused on possible interactions between bee pathogens and pesticide exposure that may be synergistic and therefore particularly harmful (James and Xu, 2012; Collison *et al*., 2016; Sánchez‐Bayo *et al*., 2016; Al Naggar and Baer, 2019; Feldhaar and Otti, 2020). A synergistic interaction can be defined as occurring when two or more stressors combine to have effect significantly greater than their additive effects. In contrast, an additive (subtractive) effect, or null interaction, is defined when the cumulative effect of two or more factors is not different to the summation (or subtraction) of their separate effects (defined as ‘additive’ and ‘subtractive’, respectively). Lastly, when two or more stressors produce a biological response that is significantly less than their individual effects, their interaction is deemed antagonistic (González‐Varo *et al*., 2013; Piggott *et al*., 2015; Maher *et al*., 2019). Across the few studies that have investigated the effect of exposure to neonicotinoids (imidacloprid, clothianidin and thiacloprid) on viral load, pesticide concentrations of ≥ 1 ppb were shown to result in increased viral titres in honey bee larvae (e.g. Doublet *et al*., 2015), suggestive of an additive effect. Recently, it has been shown that chronic exposure to FPF affects bees well beyond immediate exposure and is associated with an increased intensity of infection with *Nosema ceranae* (Al Naggar and Baer, 2019). However, potential interactive effects of the novel nAChRs insecticides FPF and SULF with other pathogens, specifically viruses such as DWV and its genotype variants, on adult bees have not yet been studied.

One means of quantifying the response of a host to a pesticide or a viral challenge is through a change in its gene expression (Aufauvre *et al*., 2014; Christen *et al*., 2018). Such studies can also help to highlight the molecular mechanisms by which hosts mount a defence against pathogens and how a pathogen evades those host defence responses (e.g. Galbraith *et al*., 2015; Doublet *et al*., 2017). Responses of honey bees to pesticide and viral challenge have been suggested to be underpinned by a common molecular pacemaker (Nazzi *et al*., 2012; Di Prisco *et al*., 2013), providing a mechanistic explanation for synergistic pesticide–pathogen interactions in *A. mellifera*.

Here, using a controlled and fully crossed laboratory experimental design, we tested the effects of chronic exposure to field‐realistic sublethal concentrations of two novel pesticides (FPF or SULF) and three viral pathogens (BQCV, DWV‐A or DWV‐B), individually and in combination, in order to identify their relative impacts as well as potential interactions (i) on the survival of individual honey bee workers, (ii) on pathogen load and (iii) on host expression of key innate immunity and detoxification genes. Our hypothesis was that we would detect a synergistic interaction between pesticide and pathogen, underpinned by a common host gene expression response.

## Results

### Effects on survival

**Fig. 1:**
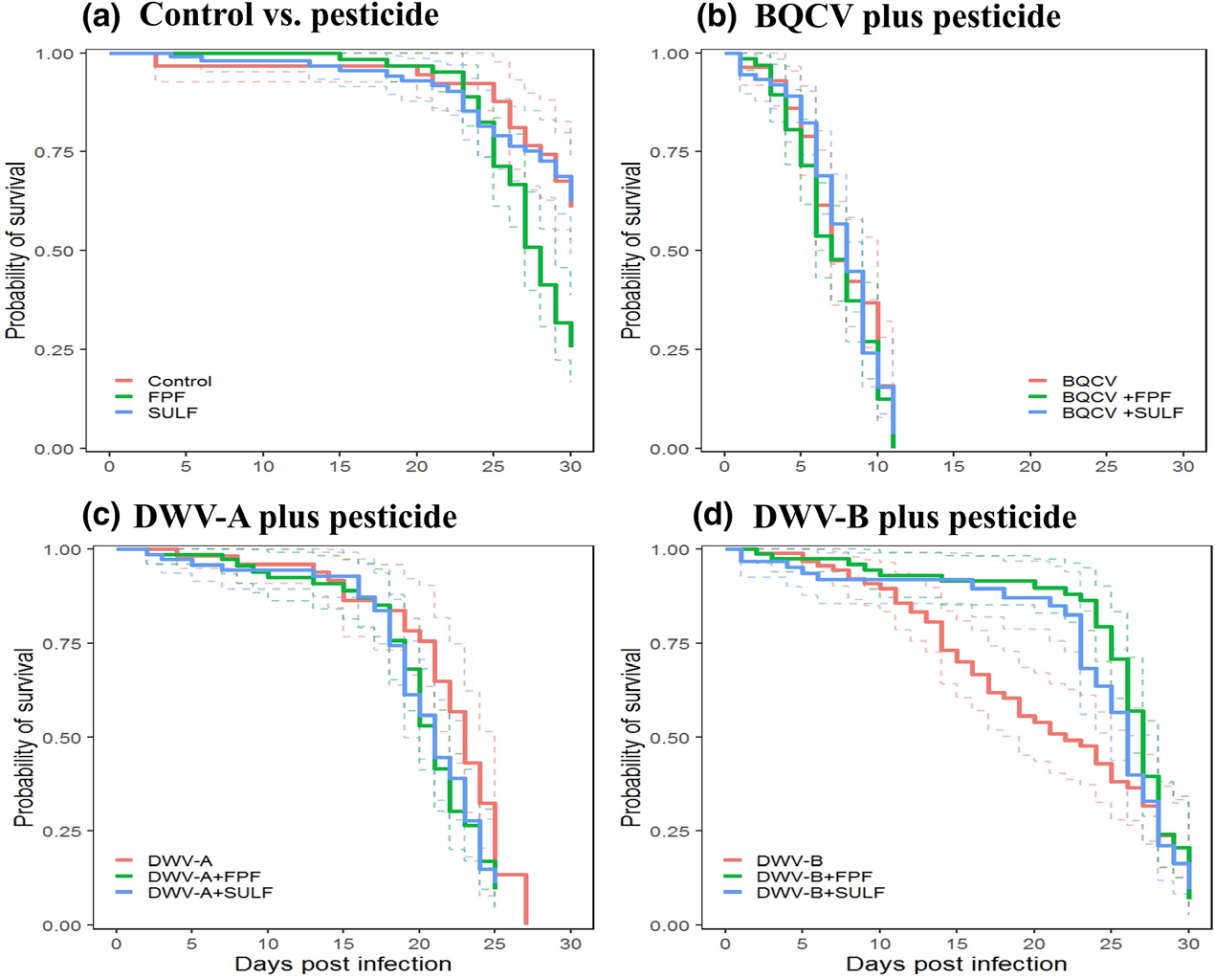
Kaplan–Meier survival curves (solid coloured lines) in days post‐infection and 95% CIs for each fitted curve (dashed coloured lines) of the impact of pesticides (FPF or SULF) and RNA viruses (BQCV, DWV‐A or DWV‐B), alone and in combination, on adult honey bees. Bees (*n* = 30 bees per cage, *n* = 3 cages per treatment) were injected with 1 µl of viral inoculum containing 10^7^ BQCV, DWV‐A or DWV‐B and then fed with sublethal concentrations of either FPF (4.300 µg ml^−1^) or SULF (0.047 µg ml^−1^) pesticides, or a control solution. Pathogens were inoculated once at day 0 while pesticides were fed *ad libitum* across the experiment; (A) honey bees treated with either FPF or SULF; (B) honey bees treated with BQCV alone and with either FPF or SULF; (C) honey bees treated with DWV‐A alone and with either FPF or SULF; (D) honey bees treated with DWV‐B alone and with either FPF or SULF. Double treatments (pesticide + virus) in (B), (C) and (D) were all statistically non‐significant compared to virus treatments alone. For statistical details, see Table S1.

We exposed adult honey bees in the laboratory to food contaminated with sublethal concentrations of either FPF or SULF for 30 days, well beyond the International Commission for Plant Pollinator Relationships (ICPPR) standard 10‐day test duration (OECD, 2017). Exposure to FPF significantly reduced survival of bees compared to non‐exposed control bees (*P* < 0.05 after correction for multiple comparisons). There was no significant effect of SULF on the survival of bees at the field‐realistic concentration we provided (*P* = 0.950) (Fig. 1, Table 1).

**Table 1:** Impact of treatments with a single pesticide or a single pathogen on honey bee survival; cage was included as a random variable as it gave a better model fit (lower AIC).

Treatment	β coefficient*	SE of β coefficient (+/−)	*Z*	*P*
FPF	0.954^ab^	0.366	2.61	**< 0.05**
SULF	−0.023^a^	0.381	−0.06	0.950
BQCV	5.074^d^	0.472	10.76	**< 0.001**
DWV‐A	2.220^c^	0.383	5.79	**< 0.001**
DWV‐B	1.754^bc^	0.349	5.02	**< 0.001**

Model‐averaged β coefficients (standardized effect size of the hazard, where higher β indicates higher risk of death) of the five variables: pesticides (FPF, SULF) and pathogens (BQCV, DWV‐A, DWV‐B) obtained from a Cox proportional hazard model in comparison with control. In bold are treatment effects that were significantly different from control by *post hoc* Tukey tests (with Bonferroni correction for multiple comparisons).

* Different lower case letters following β show significant differences among treatments (*P* < 0.05, *a posteriori* Tukey test with Bonferroni correction for multiple comparisons).

As expected, bees also had significantly lower rates of survival when inoculated with honey bee RNA viruses (BQCV: *P* < 0.001; DWV‐A: *P* < 0.001; DWV‐B: *P* < 0.001) compared to non‐infected control‐injected bees (Fig. 1, Table 1). We also found that the survival of bees inoculated with BQCV was significantly lower compared to both DWV‐A‐ and DWV‐B‐infected bees (Table 1, Fig. S1). Whereas all BQCV‐infected bees survived a median of 7 days post‐inoculation (p.i.), DWV‐A‐ or DWV‐B‐infected bees survived a median of 23 and 22 days p.i., respectively, and did not differ in survival (*P* = 0.654 (before correction for multiple comparisons), Table 1, Fig. S1).

To test for interactions between pesticides and viruses on the survival of bees, we compared the effect of these stressors in combination (‘double treatments’, e.g. ‘BQCV plus FPF’) with the effect of the stressors separately (‘single’ treatments, e.g. ‘BQCV’ or ‘FPF’). All six virus–pesticide double treatments were non‐significant in comparison to virus‐only treatments, indicating a lack of synergistic or antagonistic virus–pesticide interactions in our study; of the six comparisons between survival of virus + pesticide versus virus treatments, three were additive and three were subtractive (Fig. 1, Table S1). For one double treatment, FPF + DWV‐B, the impact of virus on survival seemed lower when bees were also treated with pesticide (Fig. 1), but differences were not significant (Table S1).

From the perspective of pesticide treatments, additionally inoculating with virus increased mortality (Table S1), all virus + pesticide versus pesticide comparisons were positive, five of six significantly so. This result is hardly surprising because pesticide alone had a small (FPF) or zero (SULF) impact on mortality whereas virus had a major impact (Table 1).

### Effects on viral load

**Fig. 2:**
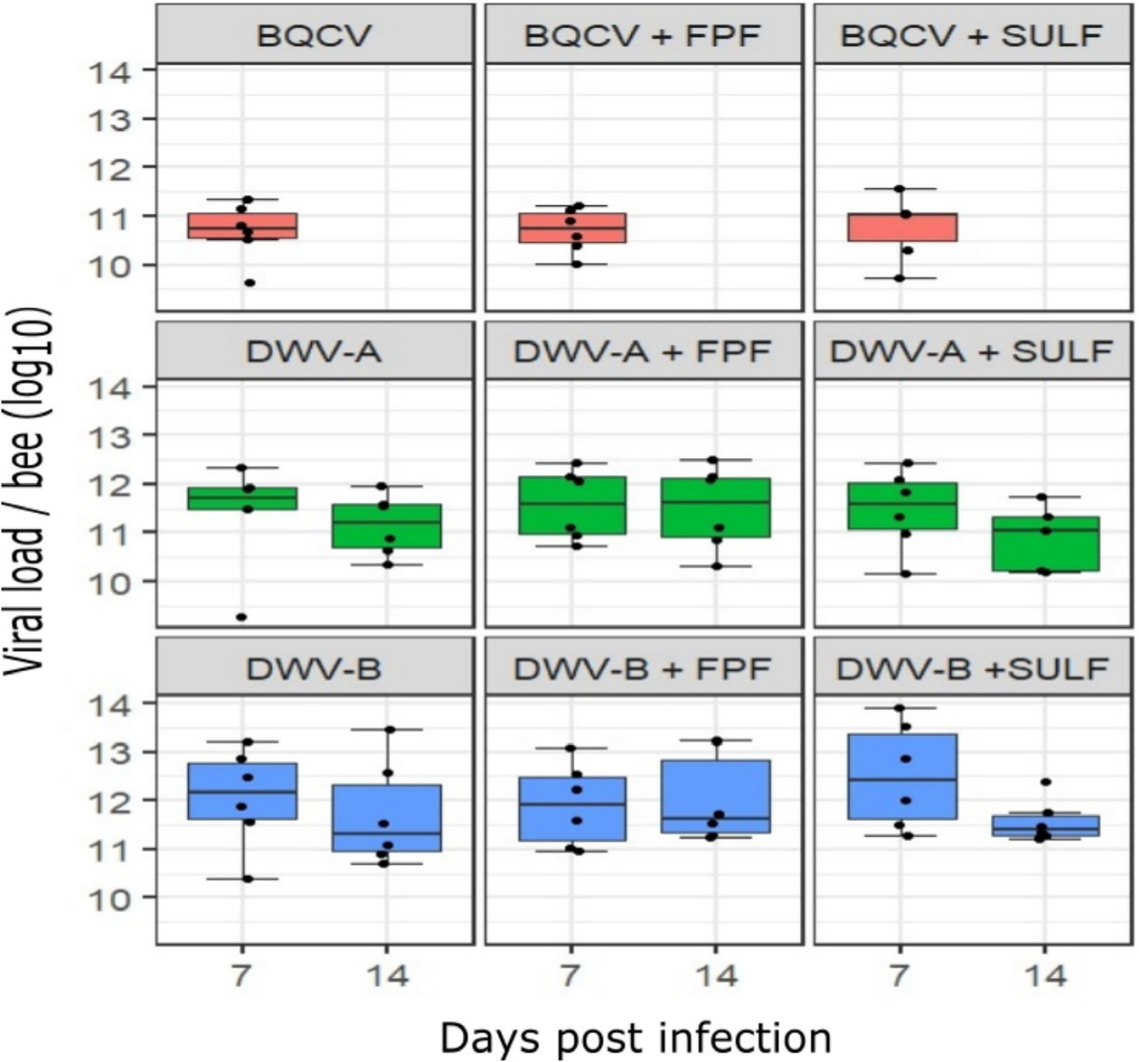
BQCV, DWV‐A and DWV‐B genome equivalents (log_10_) per adult honey bee (*n* = 6 bees per treatment and time point) at 7 and 14 days post inoculation (boxplots show median, interquartiles and 95% confidence intervals, with jittered data points). Bees were injected with 10^7^ BQCV, DWV‐A or DWV‐B then treated with sublethal concentrations of either FPF (4.300 µg ml^−1^) or SULF (0.047 µg ml^−1^) pesticides or a control solution for 30 days under laboratory conditions. There was no significant difference in viral load between virus‐exposed bees versus those co‐exposed to virus and pesticide at the two time points (LM (ANOVA Type II tests)*, P *> 0.05). Control and pesticide‐only treated bees were devoid of virus at 7 and 14 days post inoculation. For statistical details, see Table S2.

We found no significant difference in pathogen load (viral genome copy number) in bees when exposed only to virus (BQCV, DWV‐A or DWV‐B) or co‐exposed to virus and either FPF or SULF at 7 and 14 days post‐infection (LM, *P *> 0.05, Fig. 2, see Table S2 for statistical details). These results indicate that exposure to pesticides had no effect on viral load. Control and pesticide‐only treated bees were devoid of virus at 7 and 14 days p.i..

### Effects on gene expression

**Fig. 3:**
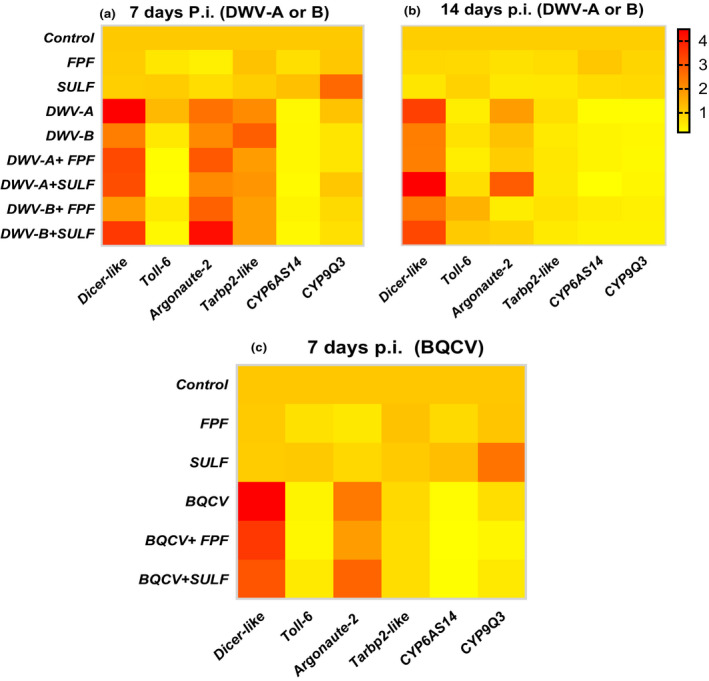
Heat map showing fold‐change in abundance of transcripts of innate immune‐ and detoxification‐related genes in adult honey bees co‐exposed to virus and pesticide at 7 and 14 days post inoculation (p.i.). Bees were inoculated with 10^7^ BQCV, DWV‐A or DWV‐B and then chronically fed with sublethal concentrations of either FPF (4.300 µg ml^−1^), SULF (0.047 µg ml^−1^) pesticides or a control solution for 30 days under laboratory conditions; (A) treatments with pesticides and DWV‐A or DWV‐B at day 7 p.i.; (B) treatments with pesticides and DWV‐A or DWV‐B at day 14 p.i.; (C) treatments with pesticides and BQCV at day 7 p.i. Colours indicate the average mRNA titres compared to average mRNA titres in control groups (*n* = 6 honey bees per treatment and time point): yellow indicates downregulation and red indicates upregulation of transcripts. Each column corresponds to one gene transcript and each row corresponds to the expression profile of a treatment (*n* = 6 bees per treatment and time point). For statistical details, see Tables S3 and S4.

When gene expression levels of bees that were singly exposed to either DWV‐A or DWV‐B were compared to those of control bees, two immune‐related (RNAi pathway) genes: *dicer‐like* and *Argonaute‐2* (AGO2) were significantly upregulated and the two detoxification genes investigated: *CYP6AS14* and *CYP9Q3* (cytochrome P450 pathway) were significantly downregulated (*P* < 0.05, Fig. 3; see Tables S3 and S4 for statistical details). In bees that were only exposed to BQCV, we also found significant upregulation of *dicer‐like* and *Argonaute‐2* (AGO2) and significant downregulation for other immune‐ and detoxification‐related genes: *tarbp2‐like*, *CYP6AS14* and *CYP9Q3* (*P* < 0.05, Fig. 3; see Tables S3 and S4 for statistical details).

When we compared the abundance of gene transcripts at 7 and 14 days post exposure across viral treatment groups, a significant interaction term DWV‐B × time was found for two genes: *Argonaute‐2* (AGO2) and *tarbp2‐like*, and a significant interaction term DWV‐A × time for *tarbp2‐like* (*P* < 0.05, Fig. 3; see Tables S3 and S4 for statistical details). This indicates that expression changes found in response to DWV‐A or DWV‐B depended on the duration of infection with these viruses.

Gene expression of the four innate immune‐related and the two detoxification‐related genes of bees exposed to either pesticide, virus or both pesticide and virus in combination did not reveal consistent pathogen–pesticide interaction effects. No significant FPF × virus interaction terms were found for any of the virus‐gene interactions investigated (Fig. 3, Table S3). The only significant interaction term we found was for SULF × DWV‐B in *dicer‐like* gene expression (*P* < 0.05; see Table S4 for statistical details), suggesting a synergistic (more than additive) interaction between these treatments on *dicer‐like* gene expression; that is, *dicer‐like* gene expression was upregulated in response to SULF + DWV‐B compared to either DWV‐B or SULF alone (Fig. S2).

## Discussion

Using a controlled experimental design that was fully crossed, we did not find evidence of synergistic interactions between either FPF or SULF insecticides and three common viral pathogens of honey bees (DWV‐A, DWV‐B and BQCV) on honey bee survival and viral load, and only one interactive effect on gene expression.

With regard to individual stressors, we found a significant reduction in survival of bees that were chronically fed on FPF at field‐realistic concentrations. Chronic exposure of adult honey bees to FPF for 30 days, instead of the standard ICPPR 10‐day test (OECD, 2017), revealed a delay in toxicity since most of the toxic effects of FPF on bee survival were observed after 18–20 days. This might reflect an accumulation of pesticide in the insect body. Under natural conditions, honey bees could be exposed to higher concentrations of these pesticides in other bee matrices like pollen pellets, bee bread, wax or royal jelly (Mullin *et al*., 2010; U.S. EPA, 2014, 2016; Böhme *et al*., 2018) or/and by dust deposits abraded from treated seeds during sowing (Schnier *et al*., 2003; Tapparo *et al*., 2012) and by contaminated water puddles (Samson‐Robert *et al*., 2014). Further research is therefore required to quantify effects of observed FPF‐induced mortality over longer timespans in the field, not only on individual honey bees but also at the colony level.

Standard methodologies currently require chronic toxicity testing over a 10‐day span (OECD, 2017). This duration seems adequate for testing pesticides with high to moderately acute toxicity for bees. The delay in toxicity of FPF on bee survival, which we observed here, would not however have been detected over a duration of ten days of standard testing. Our findings are also in line with earlier studies that reported a delay in toxicity of some pesticides such as imidacloprid (Dechaume Moncharmont *et al*., 2003), thiacloprid (Doublet *et al*., 2015) and boscalid (Simon‐Delso *et al*., 2018) in bees. A time‐to‐death approach may be more appropriate for determining the risk of chronic exposure to a pesticide, specifically when pesticides are tested at low, sublethal concentrations (Simon‐Delso *et al*., 2018), as in our experiment. All bees that had been exposed to pathogens, singly or in combination with pesticides, died by day 30, at which point we stopped the experiment. Mortality in control cages by day 30 was only ca. 40% (Fig. 1a); we were not therefore able to perform a robust time‐to‐death analysis, which requires minimally 50% mortality in control treatments (Sánchez‐Bayo, 2009; Sánchez‐Bayo and Tennekes, 2020).

Although we noticed that co‐exposure to either FPF or SULF and DWV‐B increased the survival of bees relative to DWV‐B alone (Fig. 1d), differences were not significant, suggesting a subtractive but not an antagonistic effect of pesticide on viral‐induced mortality. Bees concurrently exposed to a bacterium *(Enterococcus faecalis*) and the pesticide thiacloprid similarly had significant higher survival rates 11 days post exposure than controls (Dickel *et al*., 2018). Though Siviter *et al*. (2020a, 2020b,2020a, 2020b) found an additive impact of SULF exposure and *Nosema bombi* inoculation on bumble bee larval mortality, they found a subtractive effect of the combined pesticide–pathogen treatment on larval growth. Subtractive or even antagonistic impacts of a combined pesticide–pathogen treatment could be because the detoxification of insecticides can lead to physiological modifications that can counteract the effect of pathogens and parasites on the host (Rivero *et al*., 2010). This possibility deserves further scrutiny, though clearly it speaks against synergistic pathogen–pesticide interactions.

We found that chronic exposure of bees to either FPF or SULF had no effect on the viral load of both DWV genotypes (A and B) and of BQCV, which might explain why we did not detect a synergistic virus–pesticide effect on bee survival. Exposure to pesticides has sometimes been associated with an increased pathogenic load; a handful of studies have reported high viral titres induced by pesticide exposure in both laboratory tests with individual honey bees (Di Prisco *et al*., 2013; Doublet *et al*., 2015) and in field tests with whole colonies (Locke *et al*., 2012). Other studies have reported no direct effect of pesticides on viral load in either individual‐level or colony‐level tests (Boncristiani *et al*., 2012; Coulon *et al*., 2018; Osterman *et al*., 2019). Indeed, the pesticides fipronil and thiacloprid have been shown to reduce bee pathogen load (Vidau *et al*., 2011). This lack of consistency in response of bees to combined exposure of pesticide and pathogen suggests the effect of a pesticide on viral load is idiosyncratic and context‐dependent. For example, DWV titres briefly increased immediately after acaricide treatment with *tau*‐fluvalinate compared to untreated colonies, although this effect was not observed for BQCV and *Sacbrood virus* (SBV) (Locke *et al*., 2012). Here, we also found no direct effect of FPF on viral load, although high intensities of infection of another pathogen, *Nosema ceranae*, have been recorded in bees exposed to FPF at similar concentrations to those we used here (Al Naggar and Baer, 2019). We therefore proposed that interactive effects between pesticides and pathogens on bees could be pathogen‐ or pesticide‐specific and might depend on several factors, such as the dose of either the pesticide or the pathogen, timing of pesticide exposure, age of bees, season, life stage and genetic origin of the bees.

BQCV is currently considered a benign (low virulence) viral pathogen of adult honey bees compared to other viruses such as DWV, possibly because its mode of horizontal transmission is primarily direct, through feeding (Bailey and Woods, 1977; Chen *et al*., 2006). It has, however, been found at high prevalence and titre in collapsing colonies (Mondet *et al*., 2014), suggesting that its mode of transmission may not only be direct via ingestion but may also include indirect, vector‐mediate transmission, for example by varroa mites. Recently, we (Al Naggar and Paxton, 2020) investigated the effect of mode of horizontal transmission of BQCV either by feeding (representing direct transmission) or by injection into the haemocoel (analogous to indirect or vector‐mediated transmission) on viral virulence in individual adult honey bees; injecting BQCV directly into haemolymph in the haemocoel resulted in far higher mortality as well as increased viral titre compared to inoculation by feeding (ingestion). Here, we found that BQCV was more virulent than both DWV‐A and DWV‐B when inoculated by injection, regardless of pesticide exposure. This is consistent with our earlier findings (Al Naggar and Paxton, 2020) and suggests that BQCV may pose a future threat to honey bees and apiculture if BQCV transmission becomes primarily vector‐mediated.

Effective immune defence is likely central to honey bee health and colony survival. Individual immunocompetence can be weakened by environmental factors such as pesticides that may render honey bees more vulnerable to parasites and pathogens (Di Prisco *et al*., 2013; Al Naggar and Baer, 2019). Here, we only observed subtle interactive effects of pesticides and pathogens on the expression of one innate immune gene of honey bees; there was a significant SULF × DWV‐B synergistic interaction (upregulation) on *dicer‐like* gene expression. These findings are consistent with the lack of consistent interactive effects of pesticides on viral load that we also found. Therefore, our results do not support the hypothesis that additional stressors (e.g. exposure to pesticides) with a potential negative impact on antiviral immunity have the ability to boost unregulated viral replication (cf. Nazzi and Pennacchio, 2018).

Antiviral responses in insects appear to be mediated by two primary pathways, one involving JAK‐STAT (Janus kinase‐signal transducers and activators of transcription) and the other involving the RNA interference (RNAi) pathway, though the latter is better characterized in insects (Merkling and van Rij, 2013). The RNAi pathway functions by cleaving double‐stranded RNA (dsRNA) into small fragments, which are used to target endogenous mRNA transcripts or exogenous virus with the same sequence, and thus prevent translation of mRNA into protein and ultimately decrease the activity of the particular gene, or destroy the virus. Here, we found a pattern of upregulation of RNAi pathway‐related genes: *Argonaute‐2* (AGO2) and *dicer‐like*, that was similar in all virus‐inoculated bees, irrespective of whether or not they were exposed to pesticides. Our findings are consistent with earlier studies which reported significant upregulation for multiple RNAi pathway‐related genes, including *Argonaute‐2* (AGO2) and *dicer‐like*, in honey bees in response to acute infection with Israeli acute paralysis virus (IAPV) (Galbraith *et al*., 2015). It also supports previous studies, demonstrating that the RNAi pathway plays an important role in mediating antiviral responses in insects, including in honey bees (Maori *et al*., 2009; Liu *et al*., 2010; Desai *et al*., 2012; Flenniken and Andino, 2013).

In BQCV‐inoculated bees, we found significant downregulation of another gene linked to the RNAi pathway: *tarbp2‐like*, whether or not bees were exposed to pesticide. This interesting result could be one of the reasons why all BQCV‐infected bees in the current study died very quickly, at 11 days p.i. compared to 26 and 30 days for DWV‐A and DWV‐B‐inoculated bees, respectively (Al Naggar and Paxton, 2020).

We also found a significant downregulation of the two cytochrome P450 detoxification genes: *CYP6AS14* and *CYP9Q3*, in virus‐only inoculated bees, regardless of viral target and whether or not bees had been treated with pesticide. That downregulation of these genes did not translate into lowered survival of bees treated with pesticide and virus (compared to virus alone) is surprising. Further research is needed to investigate in greater detail the molecular mechanisms of honey bee detoxification and how gene expression relates to the ability of an individual honey bee to withstand insecticidal challenge.

## Conclusions

Here, we investigated for the first time the potential interactive effects of chronic exposure of honey bees to two novel nAChRs insecticides: flupyradifurone and sulfoxaflor, and three common honey bee viral pathogens: BQCV, DWV‐A and DWV‐B. Though FLU and pathogens reduced host survival, co‐exposure to either FPF or SULF insecticides and viral pathogens did not have a synergistic effect on honey bee survival or viral load. The SULF + DWV‐B treatment, though, led to higher *dicer‐like* gene expression. Importantly, we observed a significant reduction in the survival of bees that were chronically fed with FPF for 30 days. Our data additionally support the role of RNAi pathways in honey bee antiviral responses. Despite the reported harmful effects of sulfoxaflor on bumble bee colony fitness, our data suggest that this insecticide may pose a lesser risk to honey bees at the concentrations and endpoints we assessed.

## Experimental procedures

### Honey bees

Three colonies of *A. mellifera carnica* maintained in the General Zoology apiary at Martin Luther University Halle‐Wittenberg, Germany, were used from June to August 2019. They had been treated to control *Varroa destructor* mites with Bayvarol^®^ strips (Flumethrin, 6.61 g/strip, Bayer Vital GmbH GB, Germany) in November 2018. Prior to any research activities, colonies were inspected visually for *V. destructor* mites and by quantitative real‐time PCR (qPCR) for seven common viral targets: *Acute bee paralysis virus* (ABPV), DWV‐A, DWV‐B, BQCV, *Chronic bee paralysis virus* (CBPV), *Sacbrood virus* (SBV) and *Slow bee paralysis virus* (SBPV), using the primers listed in Table S5 and methods described in Tehel *et al*. (2019). *Varroa destructor* mites were not seen and viruses were not detected in colonies at a cycle (Ct) of 35, which is a threshold that minimizes the rate of false positives (de Miranda *et al*., 2013).

### Pesticides

We used analytical grade flupyradifurone (Sigma‐Aldrich, catalog# 37050‐100MG, Germany) and Sulfoxaflor (Ms scientific, catalog# 12883‐10MG, Germany). We dissolved FPF or SULF in ddH_2_O to obtain stock solutions with a concentration of 1μg/μl, which were stored at −20°C to avoid degradation; we did not use acetone as solvent because both compounds are easily soluble in water at the stock concentrations (FPF: 3200 mg l^−1^ at 20°C; SULF: 809 mg l^−1^ at 25°C). The feeding solutions were prepared by diluting the stock solution with 50% (w/v) aqueous sugar (sucrose) solution (hereafter: sugar water). Feeding solutions were provided *ad libitum* to the bees at the beginning of the experiment and renewed each 24 ± 2 h. These dilutions were freshly prepared every 2 days from the stock solutions, were tightly wrapped with aluminium foil to prevent light degradation and stored at 6 ± 2°C to minimize degradation of the active ingredient. When fed to bees, feeding solutions did not show signs of precipitation at any time. Feeding solutions therefore contained either pure sucrose (negative control treatment), flupyradifurone (FPF treatment; 0.0043 μg FPF μl^−1^) or Sulfoxaflor (SULF treatment; 0.000047 μg SULF μl^−1^).

Sulfoxaflor (SULF) breaks down quickly in soil but is highly persistent in water, with a half‐life of 37 days to more than a year (U.S. EPA, 2013). It takes about 8–12 days for 90% of the pesticide to dissipate from pollen and nectar (U.S. EPA, 2019). Sivanto (FPF) breaks down slowly and has been found in nectar and honey stored in wax combs for up to five months, and in nectar collected by foragers for more than two weeks (winter oil seed rape fields; U.S. EPA, 2014). Therefore, bees could be exposed to these pesticides for a long time in the field and could also be exposed to higher concentrations in other bee matrices or by dust deposits abraded from treated seeds during sowing and by contaminated water puddles (Krupke *et al*., 2012; Samson‐Robert *et al*., 2014; Azpiazu *et al*., 2019).

To simulate natural exposure to insecticides, we employed a worst‐case exposure scenario for 30 days with sublethal and field relevant concentrations of FPF and SULF to test for cumulative toxicity. Worst‐case field‐realistic concentrations of FPF (4.30 ppm) or SULF (46.97 ppb) found in nectar were given chronically *per os* to bees via sugar water. We based our dosages on Environmental Protection Agency (EPA) data that reported the residue levels of FPF in nectar and pollen of several plants such as oilseed rape (4.3 ppm in nectar and 21.0 ppm in pollen), apple pollen (39 ppm) and blueberry pollen (68 ppm) (U.S. EPA, 2014). Reported residue levels of SULF ranged between 5.41 and 46.97 ppb in nectar and between 50.12 and 510.95 ppb in pollen of sulfoxaflor‐sprayed cotton across an 11‐day period (U.S. EPA, 2016). Our pesticide exposure levels of FPF or SULF were slightly more conservative (i.e. lower) than many reported field exposures, being an order of magnitude lower than the residues found in pollen and also substantially lower than published LD_50_ estimates (Glaberman and White, 2014; U.S. EPA, 2016).

### Virus inocula and infection

Aliquots of both DWV‐A and DWV‐B were obtained from the propagated inocula of Tehel *et al*. (2019), which were retrieved from the genotype‐specific inocula of McMahon *et al*. (2016). The inocula of Tehel *et al*. (2019) and McMahon *et al*. (2016) have previously been ultradeep sequenced, revealing one nucleotide difference in the DWV‐A inoculum and three nucleotide differences in the DWV‐B inoculum between the two studies following propagation (details in Tehel *et al*., 2019), suggesting that inocula remain faithful in genotype and sequence identity during propagation. Aliquots of BQCV inocula were obtained from the propagated inoculum of Doublet *et al*. (2015). All inocula contained only the viral target and had no detectable contamination with other common honey bee RNA viruses, as determined by reverse transcription quantitative PCR (qPCR) using methods and the primers described above (Table S5).

To inoculate bees, viral inocula were first diluted in 0.5 M of cold potassium phosphate buffer (PPB) (pH 8.0) to final concentrations of 10^7^ genome equivalents per µl, as quantified by qPCR. Then, 1 µl of viral inoculum containing 10^7^ of DWV‐A, DWV‐B or BQCV was injected directly into a bee’s haemolymph between its second and third abdominal tergites using a Hamilton syringe (hypodermic needle outer diameter: 0.235 mm), simulating natural transmission by *Varroa* mites of DWV‐A, DWV‐B and potentially of BQCV (Chen *et al*., 2006; Al Naggar and Paxton, 2020).

### Exposure to pesticides and honey bee RNA viruses

Combinations of FPF or SULF pesticides and honey bee RNA viruses (DWV‐A, DWV‐B or BQCV) were tested using a fully crossed laboratory experimental design. For each of our three honey bee colonies, we transferred a single frame with sealed worker brood to an incubator kept at 34°C and 80% relative humidity (RH) overnight. The next day we collected 270 newly emerging bees per colony, and then, we randomly assigned them to different treatments. To infect bees, 1 µl of viral inoculum containing 10^7^ of DWV‐A or DWV‐B or BQCV was injected into a bee’s haemocoel. Treatments without viruses were injected with 1 µl of buffer (PPB) devoid of virus. We afterwards kept bees in metal cages (10 × 10 × 6 cm) containing an 8‐cm^2^ piece of organic beeswax, each with 30 newly emerged worker bees of the same treatment, and provided them with sugar water *ad libitum*, or sugar water containing FPF (0.0043 µg µl^−1^) or SULF (0.000047 µg µl^−1^). Though beeswax may itself contain pesticides (Boi *et al*., 2016), we used organic beeswax, which helps improve bee survival in cages (Köhler *et al*., 2013; McQuillan *et al*., 2014; Doublet *et al*., 2015; Fleming *et al*., 2015; Dussaubat *et al*., 2016; Kairo *et al*., 2017; Al Naggar and Baer, 2019).

Cages were placed into incubators at 30 ± 1°C and 50% RH. In total, we had 12 treatment groups, with three cages per treatment and 30 bees per cage. Mortality was recorded daily for 30 days. We collected subsamples (3 bees per cage) at 7 and 14 days post‐infection (i.e. 9 bees per treatment) and froze them at −80°C prior to quantifying viral load and gene expression, as described below. We also monitored the consumption of sugar water in each cage for the first 10 days of the experiment, which averaged 24.7 and 25.8 µl per bee per day of FPF and SULF‐laced sugar water for pesticide treatments. Across the 30 days of the experiment, this represents the cumulative consumption of ca. 3.18 µg per bee of FPF and 0.09 µg per bee of SULF, 2.65‐fold and 1.80‐fold greater than the reported LD_50_ of FPF and SULF, respectively (Glaberman and White, 2014; U.S. EPA, 2016).

### RNA extraction and detection of virus

When testing whether our honey bee source colonies were free of RNA virus infection, we collected 10 adult worker honey bees per colony from the brood nest, crushed them in a plastic RNAse‐free mesh bag (BioReba, Reinach, Switzerland) with 5 ml of RLT buffer after snap‐freezing on dry ice, and then recovered 100 µl of homogenate. Viral titres in adult worker bees of the inoculation experiments were determined by crushing whole bees individually in 1 ml of RLT‐buffer (with 1% beta‐mercaptoethanol) using a plastic pestle, of which 100 µl was used for RNA isolation. RNA was extracted from bee homogenates (6 bees per treatment) using an RNeasy mini kit (Qiagen, Hilden, Germany) following the manufacturer’s instructions in a QiaCube robot (Qiagen, Hilden, Germany). cDNA was synthesized from RNA extracts using oligo(dT)_18_ primers (Thermo Scientific, Schwerte, Germany) and reverse transcriptase (M‐MLV and Revertase, Promega, Mannheim, Germany) following the manufacturer’s instructions. For cDNA synthesis, 800 ng of RNA was used, after which the resultant cDNA was diluted 1:10 prior to use in qPCR.

Real‐time quantitative PCRs (qPCRs) were performed on a Bio‐Rad C1000 thermal cycler using SYBRgreen Sensimix (Bioline, Luckenwalde, Germany) and the primers for DWV‐A, DWV‐B and BQCV listed in Table S5. Amplification steps were as follows: 5 min at 95°C, followed by 40 cycles of 10 s at 95°C and 30 s at 57°C (including a read at each cycle). Following the qPCR, DNA was denatured for 1 min at 95°C then cooled to 55°C in 1 min, and a melting profile was obtained from 55 to 95°C at a 0.5°C increment per second; melt profiles consistently revealed a single product had been amplified and with the expected melt temperature. Absolute quantification of viruses was calculated using standards (ten 10‐fold dilutions of the respective PCR product covering the observed concentrations). To minimize the rate of false positives of viruses, a Ct of 35 cycles was set (de Miranda *et al*., 2013). Absolute values were log_10_‐transformed before statistical analysis.

### Gene expression

We used qPCR to quantify effects of pesticides and virus exposure on the expression of six key immune and detoxification genes that have previously been used for comparable studies of honey bee response to stressors (Galbraith *et al*., 2015; Hu *et al*., 2017). We used four genes with well‐documented involvement in the insect immune response, namely *dicer‐like*, *Argonaute‐2* (AGO2), *tarbp2‐like* and *toll‐6*, which are all part of the RNAi or Toll pathways (Brutscher *et al*., 2015; Galbraith *et al*., 2015). We selected an additional two genes with well‐documented detoxification activity: *CYP6AS14* and *CYP9Q3*, as representatives of the cytochrome P450 pathway (Hu *et al*., 2017).

The primers for all genes were taken from the literature (Galbraith *et al*., 2015; Hu *et al*., 2017) (Table S6). To quantify gene expression, we used the same synthesized cDNA as for viral quantification mentioned above and qPCRs were performed on the same machine following the same protocols used for viral detection, with slight modification in amplification steps (10 min at 95°C instead of 5 min in viral detection). The qPCR cycle was followed by a dissociation step to validate that only a single product had been amplified in each reaction. For each target gene, the abundance of transcripts was quantified according to the Mean Normalized Expression (MNE) method of Simon (2003), and β*‐actin* (AMActin) was used as a reference gene. We also determined primer efficiencies using standard curves of serial dilutions of cDNA. We confirmed acceptable reaction conditions for all genes, with efficiencies between 93 and 110% (Table S6).

### Statistical analysis

Survival analysis was performed with the R package *coxme* (Therneau, 2012) in R using mixed‐effects Cox proportional hazard models, with ‘cage’ as a nested random effect; models with ‘cage’ gave a consistently better model fit (lower AIC value) than models without this random effect. Right censored samples (bees removed at days 7 and 14 p.i. for analysis of viral titre and gene expression) were recorded in the dataset and incorporated in the Cox proportional hazard models.

In our first survival model, we tested all single treatments (pesticide or virus) with the control lacking pesticide or virus (with Bonferroni correction for multiple comparisons). To test for differences between single treatments, we performed linear contrasts (Tukey test) of Cox proportional hazard coefficients (hazard ratios) using the R package *multcomp* (Hothorn *et al*., 2013), with Bonferroni correction for multiple comparisons.

We subsequently tested for interactions between pesticide and virus in six separate models (one model per pesticide – virus combination) by coding the treatment ‘virus + pesticide’ as an independent, third treatment along with the other two treatments ‘virus’ and ‘pesticide’. Given the known impact of viruses in reducing honey bee survival, we then tested for interactive effects by comparing the treatment ‘virus’ with the treatment ‘virus + pesticide’; a significant linear contrast (Tukey test) was considered to represent a synergistic (significant positive contrast) or antagonistic (significant negative contrast) effect. A non‐significant linear contrast therefore reflected an additive (or subtractive) interaction between virus and pesticide (Piggott *et al*., 2015). Though use of multiple models may have elevated the Type I statistical error rate, all comparisons of ‘virus’ *versus* ‘pesticide + virus’ treatments were consistently non‐significant (Table S1), and therefore, adjustment of significance for multiple comparisons would not have altered results.

To test for treatment effects on gene expression, we log_10_ transformed data to meet the assumptions of normality and used ANOVA (Type II) tests in a linear model (LM). Pesticide exposure, pathogen infection and the day p.i. of bee sampling were used as independent, fixed factors (predictors). To test for significant effects of co‐exposure to pesticides, viruses and the day p.i. of sampling, we inspected the pathogen × pesticide interaction terms by keeping them in all models, independently of whether they were statistically significant or not. Using these linear models, we defined synergistic interactions as those in which the interaction term was significant (and positive) and antagonistic interactions as those in which the interaction term was significant (and negative). A non‐significant interaction term therefore reflected an additive (or subtractive) interaction between two stressors (Piggott *et al*., 2015).

To compare virus loads between pesticide treatments, we used ANOVA (Type II) tests in a LM, using pesticide exposure and day p.i. of sampling as independent, fixed factors. To test for significant interactive effects of exposure to pesticides and day of sampling p.i., we inspected the pesticide × time interaction terms by keeping them in all models, independently of whether they were statistically significant or not. Synergy and antagonism of interaction terms were again defined as for the analysis of gene expression data.

All statistical analyses and data visualizations were performed using R (R Core Team, 2020).

## Conflict of interest

The authors declare no conflict of interest.

## Supporting information


**Fig. S1.** Survival of honey bees inoculated with virus.
**Fig. S2.** *Dicer‐like* gene expression in honey bees co‐exposed to virus and pesticide.
**Table S1.** Statistical contrasts of survival among virus and pesticide treatments.
**Table S2.** Viral loads of honey bees co‐exposed to virus and pesticide.
**Table S3.** Effects of flupyradifurone and virus on honey bee gene expression.
**Table S4.** Effects of sulfoxaflor and virus on honey bee gene expression.
**Table S5.** PCR primers used for viral quantification.
**Table S6.** PCR primers used for quantification of honey bee gene expression.
