# Supplementary material for: The novel insecticides flupyradifurone and sulfoxaflor do not act synergistically with viral pathogens in reducing honey bee (Apis mellifera) survival but sulfoxaflor modulates host immunocompetence
Source: Microb Biotechnol. 2020 Sep 28;14(1):227–40. doi: 10.1111/1751-7915.13673 (PMC7888445; doi:10.1111/1751-7915.13673)
Supplement: Supplementary file 1 — Fig. S1. Survival of honey bees inoculated with virus. Fig. S2. Dicer‐like gene expression in honey bees co‐exposed to virus and pesticide. Table S1. Statistical contrasts of survival among virus and pesticide treatments. Table S2. Viral loads of honey bees co‐exposed to virus and pesticide. Table S3. Effects of flupyradifurone and virus on honey bee gene expression. Table S4. Effects of sulfoxaflor and virus on honey bee gene expression. Table S5. PCR primers used for viral quantification. Table S6. PCR primers used for quantification of honey bee gene expression. [file MBT2-14-227-s001.docx]

Supplementary Materials

**The novel insecticides flupyradifurone and sulfoxaflor do not act synergistically with viral pathogens in reducing honey bee (*Apis mellifera*) survival but sulfoxaflor modulates host immunocompetence**

Yahya Al Naggar^1,2^ & Robert J. Paxton ^1^

^1^General Zoology, Institute for Biology, Martin Luther University Halle-Wittenberg, Hoher Weg 8, 06120 Halle (Saale), Germany.

^2^Zoology Department, Faculty of Science, Tanta University31527, Tanta, Egypt.

Correspondence author Address:

General Zoology, Institute for Biology, Martin Luther University Halle-Wittenberg, Hoher Weg 8, 06120 Halle (Saale), Germany.

Tel.: +49-345 5526511

E-mail: [yehia.elnagar@science.tanta.edu.eg](mailto:yehia.elnagar@science.tanta.edu.eg)

Fig. S1. Survival of honey bees inoculated with virus

Fig. S2. *Dicer-like* gene expression in honey bees co-exposed to virus and pesticide

Table S1. Statistical contrasts of survival among virus and pesticide treatments

Table S2. Viral loads of honey bees co-exposed to virus and pesticide

Table S3. Effects of flupyradifurone and virus on honey bee gene expression

Table S4. Effects of sulfoxaflor and virus on honey bee gene expression

Table S5. PCR primers used for viral quantification

Table S6. PCR primers used for quantification of honey bee gene expression

Fig. S1. Survival of honey bees inoculated with virus


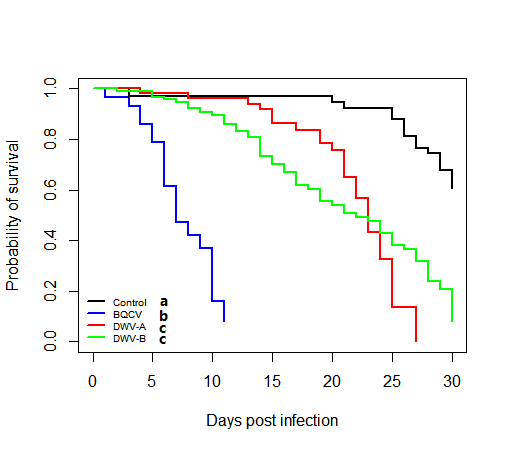


Fig. S1. Kaplan-Meier survival curves of honey bees injected with one µL of viral inoculum containing 10^7^ of either BQCV, DWV-A or DWV-B or a control solution. Different lower-case bold letters indicate statistically significant differences between treatments (Cox proportional hazards model with Bonferroni correction for multiple comparisons at *P* < 0.05; see Table 1 for statistical details).

Fig. S2. *Dicer-like* gene expression in honey bees co-exposed to virus and pesticide


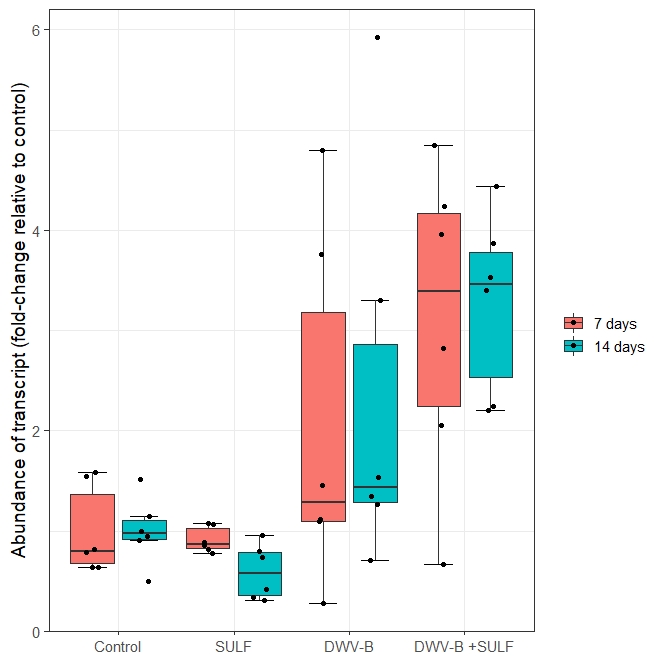


Fig. S2. Box plots (showing median, interquartiles and 95% confidence intervals, with jittered data points) of fold change in abundance of *dicer-like* (RNAi pathway) gene transcripts; expression was significantly higher in individuals co-exposed to DWV-B and SULF in adult honey bees at 7 and 14 days post inoculation (p.i.) than either DWV-B or SULF treatments alone (n= 6 bees per treatment; for statistical details, see Tables S4), and numerically higher than the sum of the SULF and DWV-B treatments. Bees were inoculated with 10^7^ DWV-B and then chronically fed with a sublethal concentration of SULF (0.047 µg/ml) pesticide, alone or in combination, or a control solution for 30 days under laboratory conditions.

**Table S1.** *ß* coefficients (standardised effect size of the hazard ± SE) of differences between treatments (pesticide+virus, pesticide, virus) based on Cox proportional hazard models of honey bee survival (one model per virus-pesticide combination). For each of the six survival models, treatments with two factors (one virus plus one pesticide) were compared to treatments with a single factor (virus or pesticide). In bold are treatment effects (interactions) that were significantly different from main effects by *post hoc* Tukey tests. The sign of the *ß* coefficient shows the direction of difference for that contrast; e.g. the *ß* coefficient for the contrast BQCV+FPF *versus* BQCV was positive (+0.197) because bees in the treatment BQCV+FPF had higher mortality than those in the treatment BQCV, though the difference was non-significant (BQCV and FPF had an additive effect on mortality).

| Treatment comparisons | | | ß coefficient | SE (+/−) | *Z* | *P* |
| --- | --- | --- | --- | --- | --- | --- |
| BQCV + FPF | vs. | BQCV | 0.197 | 0.255 | -0.770 | 0.044 |
|  |  | FPF | *5.479 | 0.772 | 7.100 | **< 0.001** |
| BQCV + SULF | vs. | BQCV | -0.009 | 0.248 | -0.034 | 0.999 |
|  |  | SULF | 3.839 | 0.741 | 6.527 | **< 0.001** |
| DWV-A + FPF | vs. | DWV-A | 0.470 | 0.454 | 1.035 | 0.554 |
|  |  | FPF | 2.315 | 0.480 | 4.824 | **< 0.001** |
| DWV-A + SULF | vs. | DWV-A | 0.370 | 0.389 | 0.950 | 0.608 |
|  |  | SULF | 2.572 | 0.437 | 5.891 | **< 0.001** |
| DWV-B + FPF | vs. | DWV-B | -0.370 | 0.365 | -1.015 | 0.567 |
|  |  | FPF | 0.413 | 0.390 | 1.061 | 0.538 |
| DWV-B + SULF | vs. | DWV-B | -0.284 | 0.257 | -1.104 | 0.510 |
|  |  | SULF | 1.421 | 0.298 | 4.765 | **< 0.001** |

*, for the comparison BQCV+FPV *versus* FPF, the coxme statistical model did not converge and therefore the *ß* coefficient and its significance were extracted from a model including treatments SULF and BQCV+SULF.

Table S2. Effects of exposure to either FPF or SULF insecticides on viral load of DWV-A and DWV-B per bee at 7- and 14-days post inoculation (p.i.). There was no significant difference in viral load between only virus-exposed bees *versus* those co-exposed to virus and pesticide at the two time points (LM (ANOVA Type II)*, P* > 0.05), and no significant difference in BQCV load between BQCV-exposed bees versus those co-exposed to virus and either FPF or SULF pesticides at 7 days p.i. (student *t*-test*, P* > 0.05) (see Figure 2).

| Source | ANOVA (Type II) | | |
| --- | --- | --- | --- |
|  | *F* value | df | *P value* |
| DWV-A | | | |
| FPF | 0.56 | 1 | 0.460 |
| time | 0.17 | 1 | 0.681 |
| FPF * time | 0.03 | 1 | 0.487 |
| SULF | 0.04 | 1 | 0.832 |
| time | 1.12 | 1 | 0.301 |
| SULF * time | 0.37 | 1 | 0.630 |
| DWV-B | | | |
| FPF | 0.06 | 1 | 0.801 |
| time | 0.07 | 1 | 0.784 |
| FPF * time | 0.37 | 1 | 0.545 |
| SULF | 0.18 | 1 | 0.670 |
| time | 2.68 | 1 | 0.116 |
| SULF * time | 0.50 | 1 | 0.484 |
| BQCV *t*-test | | | |
| FPF | 0.970 | | |
| SULF | 0.809 | | |

Table S3. Effects of flupyradifurone (FPF) and DWV-A, DWV-B or BQCV on the expression of six immunity and detoxification genes in honey bees after 7 and 14 days of exposure; significant (*P* < 0.05) terms revealed by LM (ANOVA Type II) tests are in bold. There were no significant interaction terms (FPF x viruses) for the six genes investigated (see Fig. 3 for additional details).

| ANOVA (Type II) | | | | | | | |
| --- | --- | --- | --- | --- | --- | --- | --- |
| Source | (*P*-value) | | | | | | |
|  | *Dicer-like* | *Toll-6* | | *Argonaute-2* | *Tarbp2-like* | *CYP6AS14* | *CYP9Q3* |
| DWV-A | **0.00** | 0.25 | | **0.00** | 0.61 | **0.00** | **0.00** |
| FPF | 0.17 | 0.13 | | **0.01** | 0.32 | 0.88 | 0.14 |
| Time | 0.44 | 0.26 | | 0.53 | **0.00** | 0.95 | **0.00** |
| DWV-A * FPF | 0.91 | 0.71 | | 0.52 | 0.92 | 0.29 | 0.74 |
| DWV-A * Time | 0.82 | 0.59 | | 0.09 | **0.02** | 0.17 | 0.12 |
| FPF * Time | 0.78 | 0.99 | | 0.43 | 0.57 | 0.29 | 0.60 |
| DWV-B | **0.00** | 0.83 | | **0.02** | 0.90 | **0.00** | **0.00** |
| FPF | 0.87 | 0.82 | | **0.04** | 0.93 | 0.50 | 0.46 |
| Time | 0.49 | 0.06 | | 0.07 | **0.00** | 0.96 | **0.00** |
| DWV-B * FPF | 0.32 | 0.07 | | 0.50 | 0.52 | 0.70 | 0.33 |
| DWV-B* Time | 0.25 | 0.95 | | **0.00** | **0.02** | 0.24 | 0.07 |
| FPF * Time | 0.95 | 0.64 | | 0.19 | 0.80 | 0.74 | 0.56 |
| BQCV | **0.00** | 0.17 | | **0.00** | **0.01** | **0.00** | **0.00** |
| FPF | 0.30 | 0.37 | | 0.12 | 0.68 | 0.05 | 0.11 |
| BQCV * FPF | 0.57 | | 0.87 | 0.76 | 0.62 | 0.59 | 0.26 |

Table S4. Effects of sulfoxaflor (SULF) and DWV-A, DWV-B or BQCV on the expression of 6 innate immunity and detoxification genes in honey bees after 7 and 14 days exposure; significant (*P* < 0.05) terms revealed by LM (ANOVA Type II) tests are in bold. A significant interaction term (DWV-B x SULF) was only found for *dicer-like* gene expression (see Fig. 3 for additional details).

| ANOVA (Type II) | | | | | | | | |
| --- | --- | --- | --- | --- | --- | --- | --- | --- |
| Source | | *P*-value | | | | | | |
|  |  | *Dicer-like* | | *Toll-6* | *Argonaute-2* | *Tarbp2-like* | *CYP6AS14* | *CYP9Q3* |
| DWV-A | | **0.00** | | 0.10 | **0.00** | 0.47 | **0.00** | **0.00** |
| SULF | | 0.26 | | 0.68 | 0.10 | 0.09 | 0.48 | 0.60 |
| Time | | 0.92 | | 0.23 | 0.90 | **0.00** | 0.25 | **0.00** |
| DWV-A * SULF | | 0.48 | | 0.99 | 0.38 | 0.94 | 0.91 | 0.93 |
| DWV-A * Time | | 0.15 | | 0.56 | 0.66 | 0.09 | 0.51 | 0.79 |
| SULF * Time | | 0.64 | | 0.98 | 0.97 | 0.52 | 0.85 | 0.35 |
| DWV-B | | **0.00** | | 0.23 | **0.00** | 0.99 | **0.00** | **0.00** |
| SULF | | 0.41 | | 0.91 | 0.32 | 0.39 | 0.49 | 0.75 |
| Time | | 0.92 | | 0.18 | **0.03** | **0.00** | 0.92 | **0.00** |
| DWV-B * SULF | | **0.01** | | 0.58 | 0.20 | 0.46 | 0.92 | 0.40 |
| DWV-B * Time | | 0.18 | | 0.52 | 0.06 | **0.00** | 0.68 | 0.86 |
| SULF * Time | | 0.40 | | 0.81 | 0.08 | 0.59 | 0.83 | 0.51 |
| BQCV | | **0.00** | | 0.06 | **0.01** | **0.00** | **0.00** | **0.00** |
| SULF | | | 0.13 | 0.60 | 0.44 | 0.58 | 0.30 | 0.77 |
| BQCV *SULF | 0.17 | | | 0.91 | 0.89 | 0.86 | 0.29 | 0.10 |

Table S5. List of primers used for quantification of viruses by reverse transcription followed by qPCR, and efficiency of qPCR.

| Viral target | Primer name | | Sequence | | qPCR efficiency | | Reference | |
| --- | --- | --- | --- | --- | --- | --- | --- | --- |
|  |  | |  | |  | |  | |
| ABPV | ABPV-F6548 | | TCATACCTGCCGATCAAG | | -- | | Locke *et al*. (2012) | |
|  | KIABPV-B6707 | | CTGAATAATACTGTGCGTATC | |  | |  | |
|  |  | |  | |  | |  | |
| BQCV | BQCV-qF7893 | | AGTGGCGGAGATGTATGC | | 103.3% | | Locke *et al*. (2012) | |
|  | BQCV-qB8150 | | GGAGGTGAAGTGGCTATATC | |  | |  | |
|  |  | |  | |  | |  | |
| CBPV | CBPV1-qF1818 | | CAACCTGCCTCAACACAG | | 90.2% | | Locke *et al*. (2012) | |
|  | CBPV1-qB2077 | | AATCTGGCAAGGTTGACTGG | |  | |  | |
|  |  | |  | |  | |  | |
| DWV-A | DWV-F8668 | | TTCATTAAAGCCACCTGGAACATC | | 85.6% | | Forsgren *et al*. (2009) | |
|  | DWV-B8757 | | TTTCCTCATTAACTGTGTCGTTGA | |  | |  | |
|  |  | |  | |  | |  | |
| DWV-B | VDVq-F2 | | TATCTTCATTAAAACCGCCAGGCT | | 86.0% | | McMahon *et al*. (2015) | |
|  | VDVq-R2a | | CTTCCTCATTAACTGAGTTGTTGTC | |  | |  | |
|  |  | |  | |  | |  | |
| SBPV | SBPV-F3177 | | GCGCTTTAGTTCAATTGCC | | 93.8% | | de Miranda *et al*. (2010) | |
|  | SBPV-B3363 | | ATTATAGGACGTGAAAATATAC | |  | |  | |
|  |  | |  | |  | |  | |
| SBV | SBV-qF3164 | | TTGGAACTACGCATTCTCTG | | 92.1% | | Locke *et al*. (2012) | |
|  | SBV-qB3461 | | GCTCTAACCTCGCATCAAC | |  |  | |  |
|  | |  |  |  | |  | |  |

Table S6. List of primers used for quantification of abundances of transcripts in honey bees by reverse transcription followed by qPCR, and efficiency of qPCR.

| Locus | | | Category | | Sequences | qPCR efficiency | References | |
| --- | --- | --- | --- | --- | --- | --- | --- | --- |
|  | | |  | |  |  |  | |
| *Dicer-like* | | Immunity | | | F: CCAACAGGAGCTGGAAAAAC | 103 % | Galbraith et al. (2015) | |
|  |  |  |  |  | R: TCTCCACTAAGTGCTGCACAA |  |  | |
|  | |  | | |  |  |  | |
| *TOLL-6* | | Immunity | | | F:TCCGAGGCGTCAACAGGAATCGACC | 107 % | Galbraith et al. (2015) | |
|  |  |  |  |  | R: GACAGGTCGAACGTCTCCAG |  |  | |
|  | |  | | |  |  |  | |
| *Argonaute-2* | | Immunity | | | F: TCAACAGCAGCAATCGGATA | 104 % | Galbraith et al. (2015) | |
|  |  |  |  |  | R: TTGCGGTGAACTTTGTTGTT |  |  | |
|  | |  | | |  |  |  | |
| *Tarbp2-like* (RISC) | | Immunity | | | F: AGGGTTTGCCACATGAAAGA | 102 % | Galbraith et al. (2015) | |
|  |  |  |  |  | R: AATCAGCATAACGGGCACTC |  |  | |
|  | |  | | |  |  |  | |
| Cytochrome p450 (*CYP6AS14*) | | Detoxification | | | F: TGAAACTCATGACCGAGACG | 93 % | Hu et al. (2017) | |
|  |  |  |  |  | R: AAAATTTGGGCCGCTAATAAA |  |  | |
|  | |  | | |  |  |  | |
| Cytochrome p450 (*CYP9Q3*) | | Detoxification | | | F: GTTCCGGGAAAATGACTAC | 99 % | Hu et al. (2017) | |
|  |  |  |  |  | R: GGTCAAAATGGTGGTGAC |  |  | |
|  | |  | | |  |  |  | |
| *Actin related protein 1* (*β-actin*) | | Housekeeping | | | F: ATGCCAACACTGTCCTTTCTGG | 101 % | Forsgren et al*.* (2009) | |
|  |  |  |  |  | R: GACCCACCAATCCATACGGA |  |  |  |
|  |  | | |  | |  |  |  |

References

de Miranda, J. R., Dainat, B., Locke, B., Cordoni, G., Berthoud, H., Gauthier, L., Neumann, P., Budge, G.E., Ball, B.V. & Stoltz, D. B. (2010). Genetic characterization of slow bee paralysis virus of the honeybee (*Apis mellifera* L.). Journal of General Virology, 91(10), 2524-2530.

Doublet, V., Labarussias, M., de Miranda, J. R., Moritz, R. F., & Paxton, R. J. (2015). Bees under stress: sublethal doses of a neonicotinoid pesticide and pathogens interact to elevate honey bee mortality across the life cycle. Environmental Microbiology, 17(4), 969-983.

Forsgren, E., De Miranda, J. R., Isaksson, M., Wei, S., & Fries, I. (2009). Deformed wing virus associated with *Tropilaelaps mercedesae* infesting European honey bees (Apis mellifera). Experimental and Applied Acarology, 47(2), 87-97.

Galbraith, D. A., Yang, X., Nino, E. L., Yi, S., & Grozinger, C. (2015). Parallel epigenomic and transcriptomic responses to viral infection in honey bees (*Apis mellifera*). PLoS Pathogens, 11(3), e1004713.

Locke, B., Forsgren, E., Fries, I., & de Miranda, J. R. (2012). Acaricide treatment affects viral dynamics in *Varroa destructor*-infested honey bee colonies via both host physiology and mite control. Applied and Environmental Microbiology., 78(1), 227-235.

Hu, Y. T., Wu, T. C., Yang, E. C., Wu, P. C., Lin, P. T., & Wu, Y. L. (2017). Regulation of genes related to immune signaling and detoxification in *Apis mellifera* by an inhibitor of histone deacetylation. Scientific Reports, 7(1), 1-14.
